# Influence of Aging and Salt–Alkali Coupling on the Fatigue and Self-Healing Behavior of Graphene Oxide-Modified Asphalt

**DOI:** 10.3390/ma18081808

**Published:** 2025-04-15

**Authors:** Ruixia Li, Wei Zhu, Hailong Chen, Xiao Li, Linhao Sun, Jinchao Yue

**Affiliations:** 1 School of Water Conservancy and Transportation, Zhengzhou University, Zhengzhou 450001, China; ruixiali031@163.com (R.L.); zw19990603@163.com (W.Z.); lixiao106118@163.com (X.L.); 2 Shanghai Urban Construction Maintenance Management Co., Ltd., Shanghai 200032, China; chen1543372126@163.com; 3 Zhengzhou Public Utility Investment and Development Group Co., Ltd., Zhengzhou 450001, China; sunlinhao2023@163.com

**Keywords:** GO-modified asphalt, aging, aging and salt–alkali coupling effects, viscoelastic continuum damage theory, fatigue performance, self-healing performance

## Abstract

The harsh environments in saline–alkaline areas and high-altitude regions with intense ultraviolet radiation pose great challenges to the durability of asphalt pavements. The fatigue performance of asphalt binder significantly determines the actual service life of asphalt pavements. Existing studies have predominantly focused on the impact of individual environmental factors (e.g., aging and saline–alkaline erosion) on asphalt performance, yet there remains a notable research gap in the systematic analysis of asphalt’s fatigue and self-healing behavior under coupled multi-factor interactions, particularly regarding the synergistic effects of UV aging and saline–alkaline conditions. Therefore, it is of great importance to understand the influence rules of the coupling effect of aging and salt–alkaline characteristics on the properties of asphalt materials. In this study, 70# base asphalt and GO-modified asphalt were taken as the research objects. Frequency sweep tests, linear amplitude sweep (LAS) tests, and LAS-based healing tests were conducted using a dynamic shear rheometer. The fatigue and self-healing properties of the two asphalt materials under different aging conditions and aging and salt–alkali coupling effects were analyzed based on the viscoelastic continuum damage theory. The results showed that the degree of aging can increase the stress peak of asphalt materials under small strains and also increase their stress attenuation rate. Except for short-term aging and salt–alkali effects, the aging and salt–alkali coupling effects generally further reduce the stress peaks of asphalt materials. Aging can increase the fatigue life of asphalt and increase the fatigue life attenuation rate of asphalt. The aging and salt–alkali coupling effects will reduce the fatigue life of asphalt and increase the decline rate of the asphalt fatigue life. The self-healing efficiency of asphalt is affected by the degree of aging, and the aging and salt–alkali coupling effects further reduce the self-healing efficiency of asphalt materials. This paper elucidates the influence mechanisms of intense UV irradiation and saline–alkaline environments on GO-modified asphalt, providing theoretical and practical references for its future engineering applications in harsh environmental conditions.

## 1. Introduction

Asphalt materials are widely used in high-grade pavements and urban roads due to their excellent road performance. However, prolonged exposure to natural environments subjects asphalt pavements to the combined effects of water, oxygen, ultraviolet (UV) radiation, and saline–alkaline conditions. These factors induce distresses such as cracks and potholes during service, significantly reducing pavement lifespan [1]. In saline–alkaline regions, the unique climatic and geological conditions exacerbate these challenges. Rainwater dissolves salts from the soil, forming saline–alkaline solutions that erode asphalt pavements [2]. Furthermore, high temperatures and intense radiation in these areas induce photo-oxidative aging and the salt-induced degradation of asphalt materials [3].

Extensive studies have explored the impact of saline–alkaline environments on asphalt performance. Li et al. [4] investigated the effects of water, acid, alkaline, and salt solutions on asphalt through immersion tests, revealing changes in the complex modulus and phase angle, which provided foundational insights into asphalt–saline interactions. Xu et al. [5] demonstrated that salt solutions could enhance the rheological and mechanical properties of asphalt under specific conditions, complementing Zhang’s findings and highlighting the complexity of saline–alkaline effects. Xiong et al. [6] further reported that an increased salt concentration reduces low-temperature rheological properties and weakens adhesion, though it may induce hardening under certain conditions. However, existing studies primarily focused on single-factor impacts and failed to simulate real-world multi-factorial coupling effects such as simultaneous salt erosion, UV exposure, and thermal aging.

Fatigue damage, caused by vehicular loads and environmental stressors, critically governs asphalt pavement durability. In harsh environments such as in saline–alkaline and high-altitude regions, fatigue resistance becomes even more critical. As a viscoelastic material, asphalt exhibits self-healing behavior during load-free intervals, which significantly mitigates fatigue damage. However, saline–alkaline environments may impede molecular diffusion, thereby suppressing self-healing efficiency [7]. Additionally, thixotropy, steric hardening, and nonlinear viscoelasticity further complicate fatigue performance [8]. Thus, integrating self-healing capability into fatigue life prediction models is essential for accurate pavement design.

To enhance asphalt performance, modifiers such as styrene–butadiene–styrene (SBS), styrene–butadiene rubber (SBR), and polyethylene (PE) are commonly employed. Recently, nanomaterials like graphene oxide (GO) have gained attention due to their high compatibility with asphalt, attributed to abundant carboxyl, hydroxyl, and epoxy groups [9]. Studies by Habib et al. [10] demonstrated that GO improves both high- and low-temperature elasticity. Huang et al. [11] found that GO enhances the anti-deformation and oxidation resistance of asphalt mixtures after aging. Wu et al. [12] further revealed that GO inhibits the volatilization of saturated components and forms aromatic stacking with resins, improving thermal stability. However, existing studies on GO-modified asphalt have primarily focused on investigating its rheological properties under high/low-temperature conditions while research on its fatigue and self-healing performance in complex natural environments—particularly under saline–alkaline exposure and combined high-temperature, high-humidity, and high-radiation conditions—remains insufficiently explored, lacking systematic and in-depth investigations.

In summary, although some scholars have studied the influence of single environmental factors (such as saline–alkaline erosion or ultraviolet aging) on asphalt performance, the action mechanism of multi-factor coupling effects (e.g., the synergistic effects of saline–alkaline, aging, and ultraviolet effects) on the fatigue and self-healing behavior of GO-modified asphalt in the actual service environment has not been systematically revealed. The existing literature has mostly focused on optimizing the high- and low-temperature performance of GO-modified asphalt, yet it has lacked an in-depth analysis of its damage evolution and self-healing characteristics under extreme multi-factor coupling conditions. This paper takes GO-modified asphalt and 70# base asphalt as the research objects. By simulating the complex environment of saline–alkaline solution erosion (DW, NaCl solution, Na_2_SO_4_ solution, and Na_2_CO_3_ solution), UV aging, and thermal-oxygen aging, combined with the viscoelastic continuum damage theory (VECD theory), the linear amplitude sweep (LAS) test is adopted to study the variation law of fatigue and self-healing performance of GO-modified asphalt in complex environments. This provides a key theoretical basis for the application of GO-modified asphalt in road engineering in regions with complex environments.

## 2. Materials and Methods

### 2.1. Materials

The experimental program utilized 70# base asphalt, a widely adopted pavement binder in Chinese highway engineering. Following the Test Specifications for Highway Engineering Asphalt and Asphalt Mixtures (JTG E20-2011) [13], comprehensive characterization of fundamental asphalt properties was conducted, with the obtained data systematically compiled in Table 1. Graphene oxide (GO) materials were supplied by Suzhou Danfeng Graphene Technology Co., Ltd. (Suzhou, China), and their essential physical parameters were rigorously evaluated in compliance with the international Standard ISO TS 21356:2021 “Nanotechnology-Graphene Oxide” [14]. The quantified material characteristics are presented in Table 2.

### 2.2. Sample Preparation

In this paper, GO-modified asphalt was prepared using a high-speed shearer and 70# base asphalt. GO-modified asphalts with doping amounts of 0.4%, 0.6%, 0.8%, 1.0%, and 1.2% were prepared. The 70# base asphalt was preheated in an oven at 150 °C, and then, while maintaining this temperature, a certain mass fraction of GO was added. It was sheared by a high-speed shearer at a rotation speed of 5000 rad/min for 60 min. The sheared GO-modified asphalt was placed in an oven at 120 °C for 1 h of curing and development. The basic technical indicators of the GO-modified asphalt and 70# base asphalt are shown in Table 3. Considering the penetration, softening point, and ductility of GO-modified asphalts with different doping amounts, the GO-modified asphalt with a doping amount of 1% was selected for subsequent experiments.

To simulate the complex conditions occurring in actual road surfaces, the GO-modified asphalt and 70# base asphalt were subjected to short-term aging, long-term aging, and ultraviolet aging treatments. For the short-term aging test, a rotary thin-film oven of model SYD-0609 was used. The set temperature was 163 °C, the air flow was 4000 ± 200 mL/min, the rotation speed of the turntable was 15 ± 0.2 r/min, and the aging time was 85 min. For the long-term aging, an asphalt pressure aging test chamber provided by Tianjin Gangyuan Test Instrument Factory (Tianjin, China) was employed. The set temperature was 100 °C, the pressure was 2.1 MPa, and the time was 20 h. For the ultraviolet aging test, an ultraviolet aging irradiation test chamber produced by Shaoxing Shangyu Huanke Testing Equipment Co., Ltd. (Shaoxing, China) was used, with an ultraviolet light intensity of 120 W/m^2^ in the sample area.

After the aging treatment, the samples were subjected to coupling test treatments with salt–alkali solutions (DW, NaCl solution, Na_2_SO_4_ solution, and Na_2_CO_3_ solution), and GO-modified asphalt and 70# base asphalt treated by schemes such as short-term aging and salt–alkali coupling effects, long-term aging and salt–alkali coupling effects, and ultraviolet aging and salt–alkali coupling effects were obtained.

Dynamic shear rheometer (DSR) tests were conducted using asphalt specimens with a diameter of 8 mm and a thickness of 2 mm. The asphalt, heated to a molten state, was poured into the mold and trimmed to ensure a smooth surface. After cooling, the specimens were stored in a constant-temperature chamber maintained below 5 °C. Three parallel tests were performed for each group to ensure data reliability.

### 2.3. Experimental Methods

#### 2.3.1. Frequency Sweep Test

**Figure 1:**
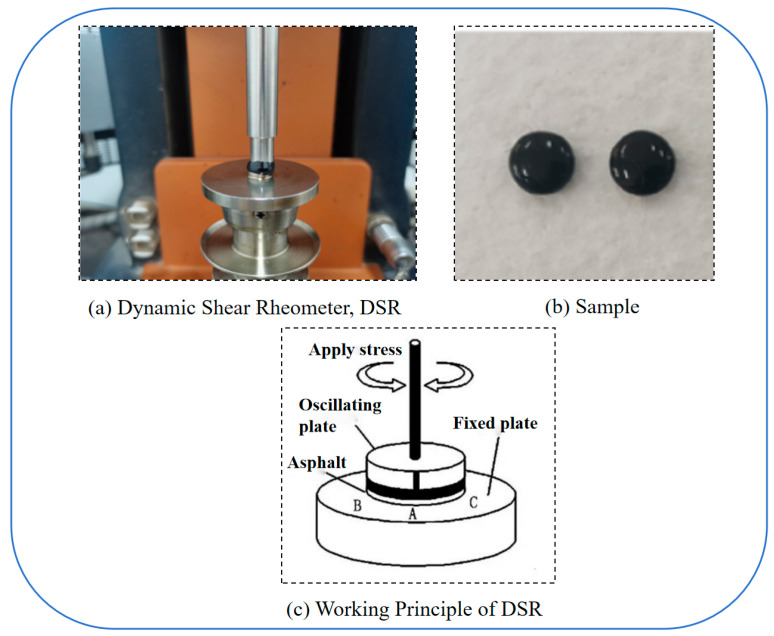
DSR test procedure.

A Dynamic Shear Rheometer (DSR) of the model Discover HR-1, which is produced by TA Instruments Inc. in the United States, was used to conduct Frequency sweep (FS) tests in accordance with AASHTO T 315 standard to characterize the viscoelastic properties of asphalt. This non-destructive testing method enables precise measurement of rheological parameters without inducing microstructural damage to the specimens [15]. The tests were conducted at 15 °C, 25 °C, and 35 °C. A carrier with a diameter of 8 mm was selected, the strain amplitude was 0.1%, and the scanning frequency range was 0.1–100 rad/s. The dynamic shear modulus, phase angle, and other parameters measured by this experiment were prepared for the subsequent fatigue tests. The DSR test procedure is shown in Figure 1.

#### 2.3.2. Fatigue Test

The fatigue test (LAS test) for GO-modified asphalt and 70# base asphalt was conducted using a dynamic shear rheometer (DSR). The test was carried out according to the AASHTO T 391 standard, adopting 8 mm diameter parallel plates. The test temperature was set at 25 °C, with the strain range selected as 0.1–30%, the strain increment as 0.1%, and the loading time chosen as 300 s [16].

The VECD theory is used to describe the variation patterns of material behavioral characteristics under dynamic loads. Since it was further developed through studies of numerous scholars, it has now become a popular research method. It integrates the continuum damage theory and viscoelastic theory to characterize the strength, stiffness of materials under high strain, and material damage conditions. Precisely for this reason, the VECD theory is widely applied to evaluate the fatigue performance of asphalt materials. Through micromechanics and continuum mechanics, the internal state changes of asphalt materials under loading are defined as damage variables, and the damage degree of asphalt materials is assessed by quantifying these variables. In this study, the VECD theory used the work potential theory to relate the modulus and damage, thereby quantifying the damage parameter *D*. Based on the stress–strain response data obtained from the LAS test and combined with the VECD theory for nonlinear data fitting [17], the fatigue damage characteristic curve (DCC) of the asphalt material was obtained. The damage degree *D* of asphalt was calculated according to Formula (1).

(1) $$D≅\sum_{i=1}^{N}{\left[\pi \gamma_{0}^{2}\left(\left|G^{*}\right|\sin\delta_{i-1}-\left|G^{*}\right|\sin\delta_{i}\right)\right]}^{\frac{\alpha }{1+\alpha }}{\left(t_{i}-t_{i-1}\right)}^{\frac{1}{1+\alpha }}$$

Here, $\gamma_{0}$ is the strain amplitude, %; $\left|G^{*}\right|$ is the complex dynamic shear modulus, Pa; δ is the phase angle, °; and *α* is the rheological parameter in the non-damaged state [18].

The DCC curve was fitted according to Formula (2).

(2) $$C=C_{0}-C_{1}D^{C_{2}}$$

Here, *C*_0_ is 1, and *C*_1_ and *C*_2_ are model fitting parameters.

The parameters *C*_1_ and *C*_2_ were obtained, and the fatigue life of the asphalt material could be calculated according to Formulas (3)–(5) and combined with the *C* value corresponding to the peak stress in the stress–strain curve in AASHTO TP 101-14 as the fatigue failure point *D_f_*.

(3) $$D_{f}={\left(\frac{C_{0}-C_{peak}}{C_{1}}\right)}^{\frac{1}{C_{2}}}$$

(4) $$A=\frac{f{\left(D_{f}\right)}^{k}}{k{\left(\pi C_{1}C_{2}\right)}^{\alpha }},B=2\alpha$$

(5) $$N_{f}=A{\left(\gamma_{\max}\right)}^{-B}$$

Here, *A* and *B* are fatigue life equation parameters, *f* = 10 Hz, *K* = 1 + (1 − *C*_2_) *α*, *C_peak_* is the *C* value corresponding to the peak stress, and *N_f_* is the fatigue life of the asphalt material. Through the above formulas, the fatigue life of an asphalt material at any strain can be predicted.

#### 2.3.3. Self-Healing Test

**Figure 2:**
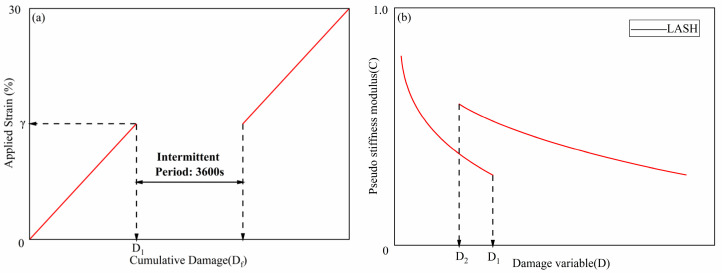
Schematic diagram of the LASH test loading principle. (**a**) Relationship between applied strain (γ) and cumulative damage (*D_f_*), with an intermittent period of 3600s. (**b**) Relationship between pseudo-stiffness modulus (C) and damage variable (D). The curve represents LASH.

Based on the VECD theory model, this study used the self-healing test based on LAS (LASH) first proposed by scholar Xie [18] in 2017. This test can greatly reduce the duration of traditional healing tests and simplify the test process. In this study, a 60 min intermittent period was introduced during the standard LAS test process. Combined with the VECD theory, the self-healing performance of GO-modified asphalt and 70# base asphalt was evaluated through the change in the damage variable parameter D before and after healing. The key to the LASH healing test is to determine the damage degree and select an appropriate healing time. Tests were carried out on GO-modified asphalt and 70# base asphalt under different aging and aging and salt–alkali coupling effects. The test process was as follows. First, a standard LAS test was carried out on the asphalt sample, and the damage parameter *D_f_* defining the fatigue failure of the asphalt based on the peak stress was calculated. In this study, 50% of the damage degree was selected as the damage degree at the start of healing, and the corresponding strain amplitude was calculated accordingly. Then, an asphalt sample treated in the same way was selected for the LAS test before healing. When the strain amplitude reached the strain amplitude corresponding to the damage degree at the start of healing, the test was stopped, and the test sample was left to heal for 1 h. Immediately after the end of the intermittent time, the standard LAS test was continued until the strain reached 30%, and the loading rate and the loaded strain amplitude were kept the same as those before healing. The loading process is shown in Figure 2a. The damage characteristic curve was obtained by using the LASH-based healing test, and its form is shown in Figure 2b.

The healing index % *H_s_* was calculated through the change in fatigue damage *D* before and after the intermittent period, as shown in Formula (6), where the calculation of *D_2_* needs to subtract the healing time [19].

(6) $$\%H_{S}=\frac{D_{1}-D_{2}}{D_{1}}\times 100\%$$

## 3. Results and Discussion

### 3.1. Fatigue Performance Analysis

#### 3.1.1. Stress–Strain Response Analysis

**Figure 3:**
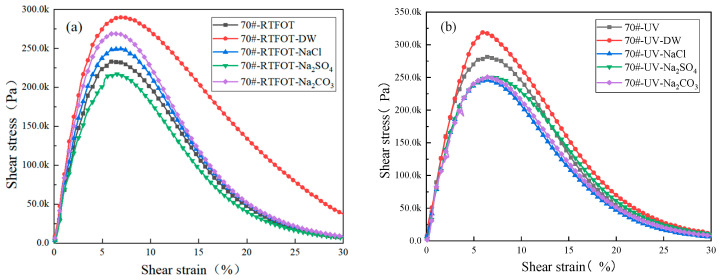

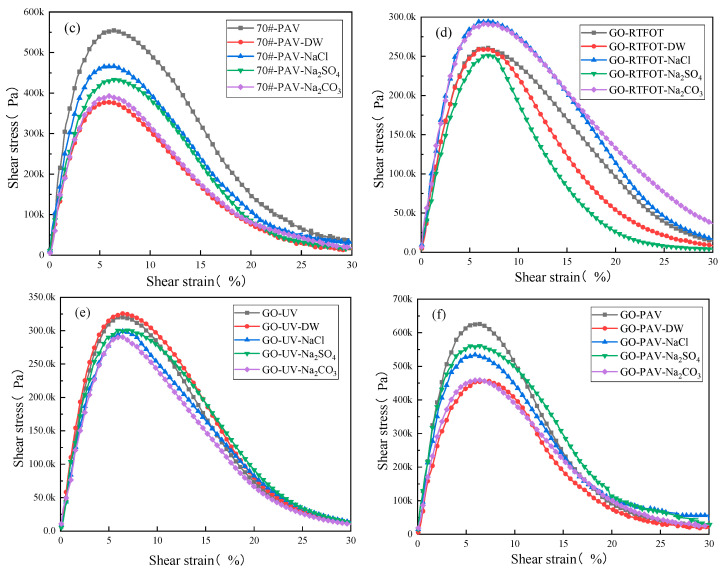
Stress–strain curves of 70# base asphalt and GO-modified asphalt.

According to the AASHTO TP101 specification, the peak of the stress–strain curve was taken as the fatigue failure point in the LAS fatigue test [20]. Based on the VECD theory, the LAS results at 25 °C were analyzed to obtain the stress–strain curves of GO-modified asphalt in complex environments, as shown in Figure 3.

Figure 3 shows that both GO-modified asphalt and 70# base asphalt exhibited a peak stress. As the strain continuously increased, the shear stress gradually increased to the peak stress and then began to decrease, indicating that fatigue damage occurred in GO-modified asphalt and base asphalt. With the deepening of the aging degree, the peak stress of both GO-modified asphalt and base asphalt gradually increased, and the influencing trend was PAV > UV > RTFOT > original sample.

Under the short-term aging and salt–alkali coupling effects, for GO-modified asphalt, sodium chloride and sodium sulfate increased the stress peak of GO-modified asphalt, having a “hardening” effect. Sodium carbonate, however, reduced the stress peak of GO-modified asphalt, weakening its deformation resistance, indicating that the short-term aged GO-modified asphalt was most sensitive to the alkaline salt environment and has a certain anti-erosion ability to sodium chloride and sodium sulfate. For base asphalt, sodium chloride and sodium carbonate reduced the strain dependence of the asphalt, with sodium carbonate having a more significant effect, while sodium sulfate reduced the stress–strain level of the base asphalt and increased its strain dependence. Thus, it can be seen that the deteriorating effect of sodium sulfate was the most obvious, accelerating the aging of the base asphalt to a certain extent.

After the ultraviolet aging and salt–alkali solution coupling effect, the saline–alkaline solutions all reduced the peak stress of GO-modified asphalt and base asphalt, indicating that the erosion of the saline–alkaline solutions reduced the deformation resistance of the asphalt and increased its strain dependence. The difference between the two was that the peak stress of GO-modified asphalt was greater than those of base asphalt, indicating that GO-modified asphalt had a greater ability to resist loads at the same strain level and a smaller strain dependence than base asphalt. Overall, for areas with ultraviolet irradiation, saline–alkaline solutions had an adverse impact on the road performance of asphalt pavements.

The GO-modified asphalt after long-term salt–alkaline coupling had greatly reduced its stress peak. The GO-modified asphalt under the sodium carbonate solution showed the worst performance, indicating that sodium carbonate had the most significant deteriorating effect on it, seriously affecting the deformation resistance of GO-modified asphalt. The stress–strain curve of the base asphalt under the long-term-salt–alkaline coupling effect was basically the same as that of GO-modified asphalt. The saline–alkaline solutions all reduced its stress peak, and the sodium carbonate solution had the most serious impact.

#### 3.1.2. Fatigue Damage Characteristic Curve Analysis

**Figure 4:**
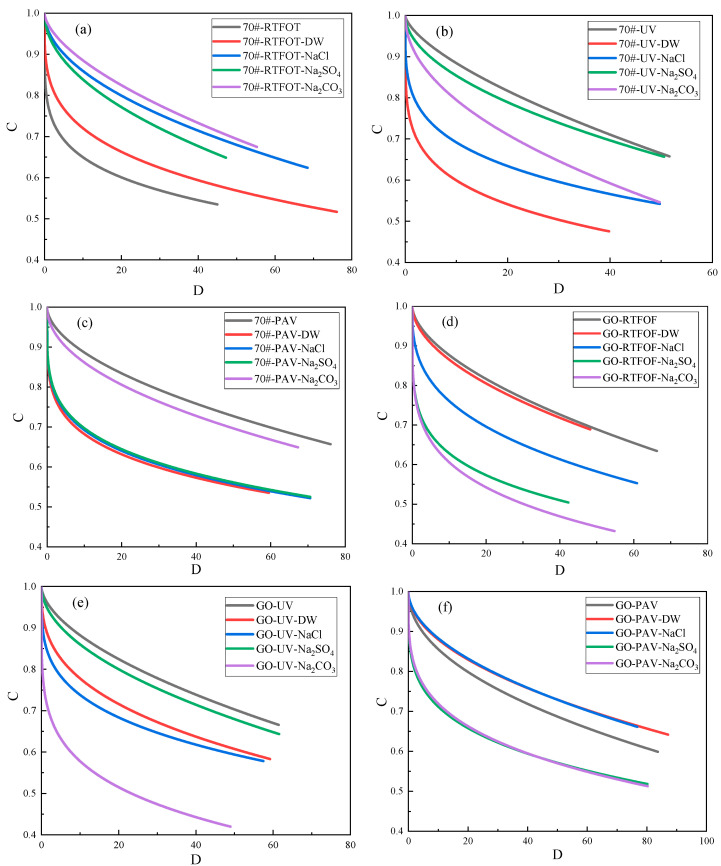
C-D curves.

Based on the VECD theory and the fatigue failure criterion, the fatigue failure point was determined. Through the LAS experimental data, the DCC curve could be drawn, which characterized the quantitative change law between the pseudo-stiffness modulus and the cumulative damage variable. The results are shown in Figure 4.

It can be seen from Figure 4 that the pseudo-stiffness modulus gradually decreased with the accumulation of the damage variable and finally reached the end-point. It was the fatigue failure point determined according to the peak theory, representing the basic fatigue damage characteristics of asphalt materials under different environmental effects. Due to the changes in the physical and chemical structures of asphalt materials caused by aging and salt–alkaline effects, different end-points occurred. The end-point value of the original GO-modified asphalt was the smallest, indicating that the cumulative damage variable was the smallest when it reached the fatigue failure point.

For GO-modified asphalt, after the short-term aging and salt–alkali coupling effects, all DCC curves were below that of the short-term aged GO-modified asphalt, and their end-points had decreased to different degrees. This indicates that the salt–alkaline environment weakened the deformation resistance of GO-modified asphalt. For base asphalt, after the short-term aging and salt–alkali coupling effects, all DCC curves were above that of the short-term aged asphalt, which meant that the salt–alkaline solution could enhance the deformation resistance of the short-term aged base asphalt, and the sodium carbonate solution had the most significant enhancement effect.

Under the ultraviolet aging and salt–alkali coupling effects, the DCC curves of GO-modified asphalt and 70# base asphalt after the action of the salt–alkaline solution were all below those under the single ultraviolet aging effect. This showed that under the same damage degree, the salt–alkaline solution accelerated the decline rate of the asphalt pseudo-stiffness modulus, indicating that the erosion of the salt–alkaline solution reduced the fatigue performance of asphalt to a certain extent. However, the sodium carbonate solution had the most serious impact on GO-modified asphalt while 70# base asphalt was most sensitive to the DW solution.

Under the long-term aging and salt–alkali coupling effects, all DCC curves were below that of the 70# base asphalt after long-term aging, indicating that the salt–alkaline environment weakened the deformation resistance of asphalt to a certain extent. The DW solution had the most significant erosion effect on 70# base asphalt. For GO-modified asphalt, the sodium chloride solution and the DW solution were above it, indicating that these two could improve the deformation resistance of asphalt to a certain extent. At the same time, the sodium carbonate solution had the most significant deteriorating effect on the long-term aged GO-modified asphalt. Preliminary analysis showed that it may have been because it was easy for the sodium carbonate solution to form voids inside the asphalt, thus reducing its fatigue resistance.

#### 3.1.3. Fatigue Life Analysis

**Figure 5:**
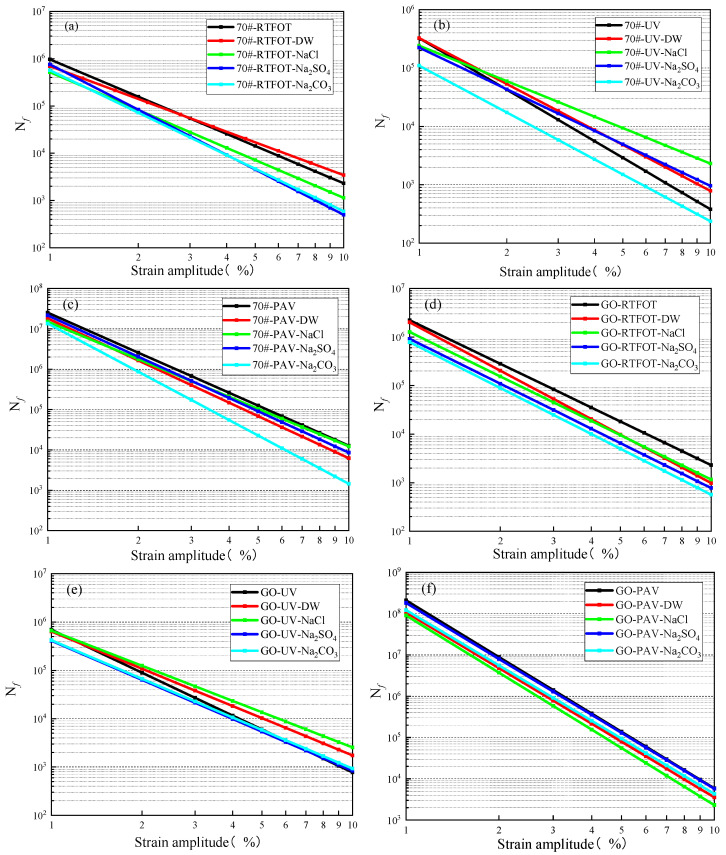
Fatigue life equations of 70# base asphalt and GO-modified asphalt.

Generally, asphalt materials under different environments have different DCC trends, and each DCC curve has different C_f_ and D_f_ values. It is usually considered that the larger the D_f_ value is, the better the durability and fatigue resistance of the asphalt material will be [21]. However, the fatigue damage rates of each asphalt material under load also vary, making it difficult to conduct a quantitative analysis of the asphalt fatigue life using the DCC curve. Therefore, it is necessary to analyze through the fatigue life equation to accurately evaluate the differences in the fatigue lives of modified asphalts under different environments. The fatigue life equations of GO-modified asphalt and base asphalt are shown in Figure 5.

Figure 5 shows that after aging, the fatigue lives of the GO-modified asphalt and the base asphalt alike increased to a certain extent. Meanwhile, the fatigue life decreased with the increase in strain, and the degree of aging could boost the material’s fatigue-resistant property to some extent. By comparing the six sets of data of the GO-modified asphalt and the base asphalt, the overall influencing trend was long-term aging > short-term aging > ultraviolet aging. However, aging also accelerated the decline rate of the fatigue life of asphalt materials. The aging process actually involved the aggregation of asphaltene micelles and the condensation of aromatic fractions, which raised the asphalt’s molecular weight [22]. In the initial stage of aging, the increase in molecular weight and the formation of more complex molecular structures boosted the material’s fatigue-resistant property to a certain extent. But as the degree of aging deepened, the brittleness of the asphalt increased. At high strain levels, the material was more likely to develop cracks and fail, thus leading to a faster decline in fatigue life.

For GO-modified asphalt, the fatigue life of GO-modified asphalt under the short-term aging/long-term aging salt–alkaline coupling effect decreased. The reason may have been that the erosion of the salt–alkaline solution accelerated the fatigue damage of GO-modified asphalt, and the ions in it destroyed the molecular structure of GO-modified asphalt, resulting in a decrease in the fatigue life. Under the ultraviolet aging and salt–alkali coupling effects, it could be clearly seen that the fatigue life of GO-modified asphalt soaked in the salt–alkaline solution increased to a certain extent under large-strain levels, and the sodium chloride solution had the most obvious improvement effect, which may have been related to the direct irradiation of ultraviolet rays.

For 70# base asphalt, under the coupling effect of short-term aging and long-term aging with salt–alkaline solutions, due to the erosion of the salt–alkaline solutions on the base asphalt, the fatigue life of the base asphalt decreased under strain levels, and the sodium carbonate solution had the greatest impact, greatly reducing the fatigue life of the base asphalt. The reason may have been that the ions in the salt–alkaline solution caused an oxidation reaction of asphalt molecules, resulting in the breakage of molecular bonds inside and the destruction of the balance of its colloidal structure, leading to a decline in the fatigue life of asphalt. Under the ultraviolet aging and salt–alkali solution coupling effect, it could be found that the sodium carbonate solution had the most significant impact on the fatigue life of the base asphalt, reducing its fatigue life, while the sodium chloride and sodium sulfate solutions could increase the fatigue life of the base asphalt under large-strain levels to a certain extent.

### 3.2. Healing Performance Analysis

#### 3.2.1. Healing Index Analysis

**Figure 6:**
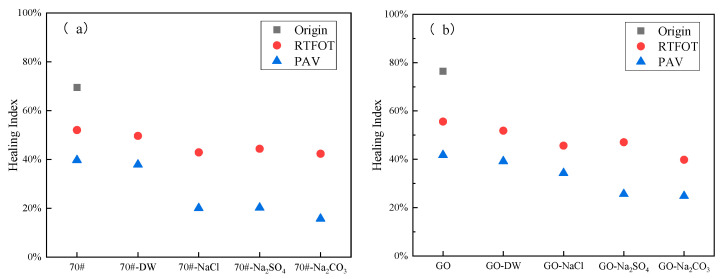
Self-healing indices of GO-modified asphalt and 70# base asphalt under different aging conditions.

Based on the VECD theory, the healing performance of base asphalt and GO-modified asphalt was evaluated by combining the LASH healing test. The healing performance was evaluated by calculating the healing index based on the damage variables before and after healing. The test results are shown in Figure 6.

The healing index quantifies the healing ability of asphalt by analyzing the relationship between the pseudo-stiffness modulus and the damage degree. The larger the value is, the stronger the healing ability of the asphalt is. It can be seen from Figure 6 that the original base asphalt and the original GO-modified asphalt had the highest self-healing rates. After the asphalt underwent short-term aging, ultraviolet aging, and long-term aging, its self-healing decreased. And the influencing effect was long-term aging > ultraviolet aging > short-term aging. The reason was that compared with short-term aging and ultraviolet aging, under long-term aging, oxygen reacted with the double bonds in asphalt molecules, generating more oxidation products, changing the molecular structure of the asphalt, thus affecting the diffusion rate of the asphalt and reducing its self-healing efficiency.

It can be seen from Figure 6 that the self-healing efficiency of GO-modified asphalt was basically better than that of base asphalt in various complex environments, indicating that GO had a positive impact on the self-healing performance of asphalt, manifested as an increase in the self-healing index of asphalt. The addition of GO molecules could form hydrogen bonds with asphalt molecules and generate intermolecular van der Waals forces, and form aromatic ring stacking with aromatics and resins, enabling GO to be stably dispersed in asphalt and form stable connections, thus improving the road performance of asphalt. At the same time, it could affect the transformation between components and enhance the self-healing diffusion movement of asphalt molecules, thereby increasing the self-healing index of asphalt. Currently, there has been no unified conclusion on the modification mechanism of GO, and further research is needed.

When the salt–alkaline and aging were coupled, the self-healing rates of both base asphalt and GO-modified asphalt declined to a certain extent. The erosion of the DW had a relatively small impact on reducing the self-healing efficiency of base asphalt and GO-modified asphalt, indicating that the DW had had little impact on the self-healing of asphalt. For base asphalt, the self-healing efficiency was the lowest under the coupling of the sodium carbonate solution and aging, indicating that the base asphalt was most sensitive to alkaline salts, and the alkaline salts had the most serious deteriorating effect on the base asphalt. For GO-modified asphalt, the sodium sulfate solution had the most serious impact on GO-modified asphalt. It was speculated that the reason was that $SO_{4}^{2-}$ affected the connection between GO molecules and asphalt molecules, thus reducing its self-healing efficiency.

#### 3.2.2. Fatigue Life Analysis Before and After Healing

With the introduction of the load intermittent period, we could clearly observe the recovery of the asphalt modulus in the LASH test, which promoted the recovery of the asphalt fatigue life. Therefore, it is necessary to analyze this. For this section, the fatigue life of the self-healed asphalt was calculated based on the VECD theory. At the same time, according to the empirical formula proposed by Mannan [23], the fatigue life coefficient after healing was calculated. A fatigue life conversion coefficient μ was introduced to quantitatively analyze the improvement effect of the fatigue life after self-healing. The calculation formula of this coefficient is shown in Formula (7).

(7) $$\mu =\frac{N_{f}(with-rest)}{{N^{\prime }}_{f}(without-rest)}$$

Here, $N_{f}$ and ${N^{\prime }}_{f}$ are the fatigue lives of asphalt calculated by the LAS test and the LASH healing test, respectively, according to the peak stress failure criterion. The calculation results of the fatigue life conversion coefficient μ are shown in Table 4.

**Figure 7:**
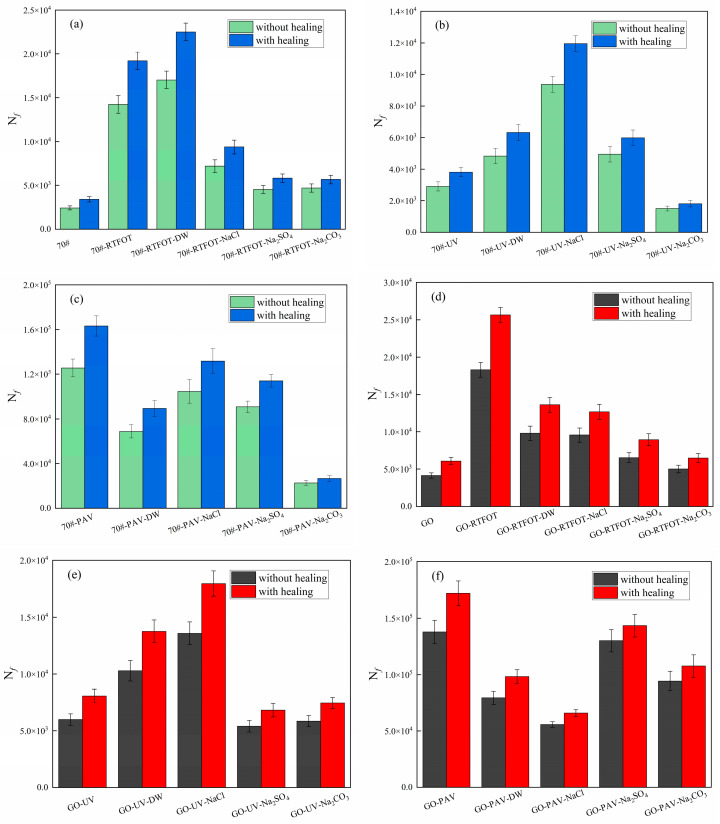
Fatigue lives before and after healing.

It can be seen from Table 4 that the μ value of the original asphalt was the highest, and the μ value decreased after aging, indicating that aging hindered the recovery of the asphalt fatigue life. With the deepening of aging, the μ value showed a downward trend, and the influencing trend was long-term aging > ultraviolet aging > short-term aging. After the aging and salt–alkali coupling effects, it could be found by comparison that the μ values all decreased, indicating that the salt–alkaline environment hindered the recovery of the asphalt fatigue life. For more intuitive display, and according to the research of domestic and foreign scholars [24], when the strain level γ=2.5% and 5%, they corresponded to the actual strain levels of thick-layer and thin-layer asphalt pavements, respectively, and when the strain level was 5%, the fatigue life of asphalt had a high correlation with the fatigue life of the mixture. Figure 7 shows the comparison of the fatigue lives of the two asphalts before and after healing at a strain of 5%.

It can be seen from Figure 7 that due to the introduction of the intermittent period, the self-healing performance of asphalt had a certain growth effect on its fatigue life. In the 60 min intermittent period, it could be seen that the fatigue lives of all asphalt samples increased to different degrees. The growth efficiency of the original sample was the highest while the growth rates of the asphalt after short-term aging, ultraviolet aging, and long-term aging decreased. The long-term aged asphalt had the worst recovery effect. The reason was that in a high-temperature and high-pressure environment, the light components in the asphalt volatilized, and the increases in hard components hindered the flow of the asphalt, suppressing the healing of micro-cracks in the asphalt, thus reducing the ability of the asphalt to withstand the secondary load.

After the aging and salt–alkali coupling effects, it could be found that the growth effect of the asphalt fatigue life was further reduced, indicating that the salt–alkaline solution also inhibited the fatigue and self-healing performance of asphalt. For the asphalt under the short-term aging and salt–alkali coupling effects, it could be seen that the fatigue life growth effect of the asphalt under the action of the sodium carbonate solution was the worst. Under the ultraviolet aging and salt–alkali coupling effects and the long-term aging and salt–alkali coupling effects, for the base asphalt, the fatigue life growth effect of the asphalt under the action of the sodium carbonate solution was the worst, while GO-modified asphalt was more sensitive to the sodium sulfate solution, resulting in a poor fatigue life growth effect.

In addition, by comparing the fatigue lives of 70# base asphalt and GO-modified asphalt after healing, it could also be found that the fatigue life growth effect of GO-modified asphalt was generally higher than that of base asphalt, indicating that GO molecules could improve the self-healing performance of base asphalt. This benefited from the compatibility of GO molecules with asphalt, enabling them to form a more stable molecular structure with asphalt molecules, thus improving the self-healing performance of asphalt and enhancing its fatigue life growth effect.

## 4. Conclusions

This paper studied the influence of different aging and salt–alkali coupling effects on the fatigue and self-healing of asphalt and analyzed it, combined with the viscoelastic continuum damage theory, expounding the change rules of the fatigue and self-healing performance of asphalt under different aging and salt–alkali coupling effects. The main conclusions are as follows:

(1) Aging increased the peak stress but accelerated the stress decline rate. Under the short-term aging and salt–alkali coupling effects, the sodium carbonate solution could significantly increase the peak stress of asphalt while the sodium sulfate solution would significantly reduce them. Under the ultraviolet aging and salt–alkali coupling effects and long-term aging and salt–alkali coupling effects, the salt–alkaline solutions had an adverse impact on asphalt, especially the sodium carbonate solution.

(2) The fatigue lives of GO-modified asphalt and base asphalt increased with the increase in the aging degree, but with the increase in the strain, the decline rate of the fatigue life of asphalt materials also increased. Under the salt–alkaline-aging coupling effect, the presence of salt–alkaline solutions reduced the fatigue life of asphalt, with the alkaline solution having the most significant impact. At the same time, it also increased the decline rate of the fatigue life of asphalt materials.

(3) Saline–alkaline solutions degraded the self-healing performance of both GO-modified and base asphalt. Under different aging and salt–alkali coupling effects, the salt–alkaline effect reduced the self-healing performance of the two kinds of asphalt, making the growth amplitude of their fatigue life decrease. The growth was the smallest under the long-term aging and salt–alkali coupling effects solution, and the asphalt life growth under the erosion of the sodium carbonate solution was the smallest, indicating that asphalt was most sensitive to the alkaline environment.

In this study, a large number of laboratory tests were carried out to investigate the fatigue and self-healing properties of GO-modified asphalt and 70# base asphalt under different aging effects and aging and salt–alkali coupling effects. However, there are still some issues that need further research. First, this paper has only focused on the research on asphalt binders. Future studies can explore the fatigue and self-healing properties of asphalt mixtures when subjected to different aging scenarios, with a particular focus on the coupling of aging and salt–alkali effects. This is of great significance for the research on the road-use performance of asphalt pavements in saline-alkali areas and high-altitude areas with strong ultraviolet radiation. Second, regarding the research on the self-healing properties of asphalt, it is necessary to further consider the influence of factors such as temperature, thixotropy, and intermittent time on the self-healing properties of GO-modified asphalt. This is conducive to improving the prediction accuracy of the fatigue life of asphalt.

## Figures and Tables

**Table 1:** Fundamental properties of 70# base asphalt.

Property	Unit	Test Result	Specification Requirement	Test Method (JTG E20-2011)
Penetration (25 °C)	0.1 mm	64.3	60–80	T0604-2011
Softening point	°C	47.5	≥45	T0606-2011
Ductility	cm	34	≥25	T0605-2011

**Table 2:** Fundamental properties of graphene oxide (GO).

Material	Technical Parameter	Measured Value
Graphene Oxide	Purity (%)	>96
	Sheet Diameter	10–50
	Thickness (μm)	3.5–7.0
	Number of Layers	6–10
	Specific Surface Area (m^2^/g)	100–300
	Appearance	Black Powder Particles

**Table 3:** Basic technical indicators of GO-modified asphalt and 70# base asphalt.

Asphalt Type	Penetration (25 °C)/0.1 mm	Softening Point/°C	Ductility/cm (10 °C)
70#	64.3	47.5	34
0.4% GO	61.3	48.1	23.2
0.6% GO	60.9	49.5	21.1
0.8% GO	59.2	50.2	20.3
1.0% GO	58.7	50.8	19.4
1.2% GO	58.4	51.0	18.9

**Table 4:** Fatigue life conversion coefficient μ of asphalt under different aging and salt–alkali coupling effects.

Asphalt Type	Original	RTFOT	UV	PAV
70#	1.407	1.349	1.312	1.299
70#-DW	-	1.321	1.308	1.298
70#-NaCl	-	1.303	1.276	1.261
70#-Na_2_SO_4_	-	1.282	1.208	1.255
70#-Na_2_CO_3_	-	1.207	1.199	1.174
GO	1.469	1.402	1.349	1.248
GO-DW	-	1.389	1.338	1.238
GO-NaCl	-	1.325	1.321	1.185
GO-Na_2_SO_4_	-	1.363	1.261	1.102
GO-Na_2_CO_3_	-	1.299	1.272	1.141

## Data Availability

The original contributions presented in the study are included in the article; further inquiries can be directed to the corresponding author.
